# Recreational Drowning Deaths in the South West of England

**Published:** 1991-09

**Authors:** Andrew Rouse

**Affiliations:** Senior Registrar in Public Health Medicine Frenchay Health Authority


### Recreational Drowning Deaths in the South West of England

**Andrew Rouse MFPHM**
Senior Registrar in Public Health Medicine
Frenchay Health Authority

### BACKGROUND

Tourism, much of which is based on the recreational use of water, is a major source of income in the South West. It is common knowledge that these activities carry the risk of drowning. This study was done in an attempt to identify where strategies aimed at prevention of recreational drownings should be concentrated. In this paper, the South West of England refers to the 5 counties Gloucester, Avon, Somerset, Devon and Cornwall.

### METHOD

The Royal Society for the Prevention of Accidents publishes an annual compendium of drowning incidents.[1] Data from these reports for the years 1987-90 were combined together and analyzed.

### RESULTS

At least 81 drowning deaths could be directly related to the recreational use of water. Information on these 81 drownings is analyzed below, and shown in Table 1. Unfortunately no information is available on the place of residence of these victims, and it is likely that many were visitors to the South West. Nearly half of the deaths occurred in Cornwall, and male victims outnumbered females. The median age of victims depends on the activity associated with drowning. The median age of swimming victims was appreciably younger (by about 15 years) then victims of other activities (median age 33 years). Not surprisingly nearly half of all deaths occur in the summer months. The table gives details on the more common activities associated with 77 of the 81 recreation drownings. Fishing, swimming and boating account for most deaths. Two deaths were associated with diving into water, one death with playing in water and one death occurred as a result of someone walking near water attempting to rescue someone in the water.

### DISCUSSION

These findings are consistent with the data reported on recreational drownings deaths in England and Wales by the Office of Population Census and Surveys,[2] and are relevant to any health promotion or educational activity aimed at reducing deaths from drowning. They suggest that such activities can be directed at a small group of the population. For instance further analysis shows that 33% (27/81) of all recreational drowning deaths occurred in Devon and Cornwall amongst males engaged in salt water activities in the months of June, July, August and September.

### FISHING

Fishing would appear to be a relatively hazardous activity. Relative risks of the various recreational activities cannot be assessed since we have no estimates of the number of person hours spent fishing as compared to other sports. However OPCS data[2] suggests that participation in swimming is 6 times more frequent than fishing. If so fishing is 5 times (6 x 21/25) more dangerous than Swimming! It is also interesting to note that two thirds of all fishing related drowning deaths occurred amongst land, not boat based fisherman.

### ALCOHOL USE

Little information is available on the role of alcohol in these recreational drowning deaths. However a review of the literature suggests that between 25-50% of such deaths are associated with alcohol use. However since the frequency of alcohol use amongst persons who do not drown whilst participating in water sports is unknown, the exact contribution of alcohol to drowning deaths remains uncertain.

### CONCLUSION

Health promotion — accident prevention strategies can be directed at specific groups, in particular fisherman, or males engaged in saltwater sports in Devon and Cornwall.

### REFERENCES

1. Drowings in the UK (1987/88/89/90). Royal Society for the Prevention of Accidents.
2. OPCS Monitors. DH4 Series (e.g. 88/3) Office of Population Census and Surveys.

**Table 1:**
**Details of Deaths Associated with Recreational Use of Water**

| | Swimming | Boating | Fishing | Surfing | Sub Aqua | Other | Total |
|---|---|---|---|---|---|---|---|
| No. Male | 20 | 17 | 21 (14 land 7 water) | 5 | 7 | 4 | 74 |
| No. Female | 5 | None | None | None | 2 | 0 | 7 |
| Median age M (in years) | 18 | 36 | 39 | 33 | 34 | — | — |
| Median age F (in years) | 23 | — | — | — | 41 | — | — |
| No. in January, February, March | 0 | 8 | 2 | 0 | 1 | 0 | 11 |
| No. in April, May, June | 4 | 2 | 6 | 1 | 7 | 0 | 20 |
| No. in July, August, September | 15 | 3 | 9 | 4 | 1 | 3 | 35 |
| No. in October, November, December | 6 | 4 | 14 | 0 | 0 | 1 | 15 |
| No. in Avon | 7 | 2 | — | — | — | 1 | 10 |
| No. in Cornwall | 8 | 5 | 13 | 5 | 4 | 2 | 37 |
| No. in Devon | 5 | 9 | 3 | — | 5 | 1 | 23 |
| No. in Gloucestershire | 1 | — | 1 | — | — | 2 | 2 |
| No. in Somerset | 4 | 1 | 4 | — | — | 0 | 9 |
| Sea | 10 | 8 | 17 (11 from land) | 5 | 9 | 1 | 50 |
| River | 4 | 8 | 1 | — | — | 1 | 14 |
| Canal | — | 1 | — | — | — | — | 1 |
| Pool | 9 | — | — | — | — | 1 | 10 |
| Lake | 2 | — | 3 (from land) | — | — | 1 | 6 |

